# Supplementary material for: Data in support of FSH induction of IRS-2 in human granulosa cells: Mapping the transcription factor binding sites in human IRS-2 promoter
Source: Data Brief. 2015 Dec 13;6:162–7. doi: 10.1016/j.dib.2015.12.001 (PMC4706562; doi:10.1016/j.dib.2015.12.001)
Supplement: Supplementary file 2 — Supplementary material [file mmc2.pdf]

Elsevier Editorial System(tm) for Data in Brief
Manuscript Draft

Manuscript Number: DIB-D-15-00526R1

Title: Data in support of FSH Induction of IRS-2 in human granulosa cells: Mapping the transcription factor binding sites in human IRS-2 promoter

Article Type: Data Article

Keywords: IRS-2, TFBS, FSH, SP1, ChIP

Corresponding Author: Prof. Rita Singh, Ph.D

Corresponding Author's Institution: Delhi University

First Author: Rita Singh

Order of Authors: Rita Singh

Abstract: Insulin receptor substrate-2 (IRS-2) plays critical role in the regulation of various metabolic processes by insulin and IGF-1. The defects in its expression and/or function are linked to diseases like polycystic ovary syndrome (PCOS), insulin resistance and cancer. To predict the transcription factors (TFs) responsible for the regulation of human IRS-2 gene expression, the transcription factor binding sites (TFBS) and the corresponding TFs were investigated by analysis of IRS-2 promoter sequence using MatInspector Genomatix software [1]. The ibid data is part of author's publication [2] that explains Follicle stimulating hormone (FSH) mediated IRS-2 promoter activation in human granulosa cells and its importance in the pathophysiology of PCOS. Further analysis was carried out for binary interactions of TF regulatory genes in IRS-2 network using Cytoscape software tool and R-code. In this manuscript, we describe the methodology used for the identification of TFBSs in human IRS-2 promoter region and provide details on experimental procedures, analysis method, validation of data and also the raw files. The purpose of this article is to provide the data on all TFBSs in the promoter region of human IRS-2 gene as it has the potential for prediction of the regulation of IRS-2 gene in normal or diseased cells from patients with metabolic disorders and cancer.

Detailed response to Reviewers:

Revised Manuscript has following changes:

The title is changed to "Data in support of FSH Induction of IRS-2 in human granulosa cells: Mapping the transcription factor binding sites in human IRS-2 promoter"

We have revised the text in all sections of the manuscript as per the DiB requirements. We have improved the abstract, specification table, value of data and the data section.

4th line is added to Transcription factor binding sites analysis section of Experimental design.

The FSH activated TFs are highlighted yellow in Table 1. Table 2 is not changed.

We have added the data on gene networks for IRS-2 as Fig. 3 and raw data as excel file in supplementary material.

All 3 figures have been combined in a single power point file (Figure 1-3-Singh R-revised)

Figure captions are given as a separate word file.

A folder containing all the raw data and the excel sheets of the tables is provided as supplementary material.

References: Reference no. 2 and 4 are added.

We have quoted the original paper(CLS-D-15-00333R1) in the reference as [2] and included the reference in the abstract.

*Manuscript
Click here to view linked References

Data in support of FSH Induction of IRS-2 in human granulosa cells: Mapping the transcription factor binding sites in human IRS-2 promoter

Surleen Kaur, G. Anjali, Priya Bhardwaj, Jyoti Taneja and Rita Singh†

Division of Molecular Endocrinology and Reproduction, Department of Zoology, University of Delhi, Delhi-110007

Email: ghrika_s@yahoo.com

†Corresponding Author

Address: Department of Zoology, University of Delhi, Delhi-110007, India

E-mail: ghrika_s@yahoo.com

Telephone: +91-8527820707, 91-11-27667985/308

Keywords

IRS-2, TFBS, FSH, SP1, ChIP

Abstract

Insulin receptor substrate-2 (IRS-2) plays critical role in the regulation of various metabolic processes by insulin and IGF-1. The defects in its expression and/or function are linked to diseases like polycystic ovary syndrome (PCOS), insulin resistance and cancer. To predict the transcription factors (TFs) responsible for the regulation of human IRS-2 gene expression, the transcription factor binding sites (TFBS) and the corresponding TFs were investigated by analysis of IRS-2 promoter sequence using MatInspector Genomatix software [1]. The ibid data is part of author's publication [2] that explains Follicle stimulating hormone (FSH) mediated IRS-2 promoter activation in human granulosa cells and its importance in the pathophysiology of PCOS. Further analysis was carried out for binary interactions of TF regulatory genes in IRS-2 network using Cytoscape software tool and R-code. In this manuscript, we describe the methodology used for the identification of TFBSs in human IRS-2 promoter region and provide details on experimental procedures, analysis method, validation of data and also the raw files. The purpose of this article is to provide the data on all TFBSs in the promoter region of human IRS-2 gene as it has the potential for prediction of the regulation of IRS-2 gene in normal or diseased cells from patients with metabolic disorders and cancer.

1. Specifications table

Subject area	*Biology*
More specific subject area	*Gene regulation and TFBS*
Type of data	*MatInspector data, figures and table*
How data was acquired	*In silico analysis of IRS-2 promoter sequence using Genomatix MatInspector software, ChIP assay, qRT-PCR*
Data format	*Raw Excel spreadsheet (.xls), filtered and analysed data*
Experimental factors	*Isolation of human GCs from the follicular fluid aspirates obtained after IVF treatment, grown in culture and FSH treated. Precipitation of protein-DNA complexes with anti-SP1 antibody and PCR*
Experimental features	*Analysis of known TFBS on IRS-2 promoter using MatInspector software, filtering the TFs that are regulated by FSH, validation of the induction of SP1 binding to IRS-2 gene promoter by FSH in human GCs by ChIP assay*
Data source location	*Delhi, India*
Data accessibility	*Data are provided with this article.*

2. Value of the data

- IRS-2 protein is an important signalling component in the regulation of metabolism in human and other organisms. However, specific transcription regulation of IRS-2 has not yet been characterized completely in the existing literature.
- This data exhibits all TFBS and the corresponding TFs that may bind human IRS-2 promoter.
- FSH stimulated TFs and activation of human IRS-2 promoter.
- The data provided in this paper would be extremely relevant for further analysis of the regulation of IRS-2 interactions especially related to cancer progression.

3. Data

In order to identify the TF responsible for the activation of IRS-2 promoter activity downstream FSH, all TFBS in the IRS-2 promoter region [3] were explored using the Genomatix MatInspector software (Fig. 1) followed by a search for TFs that are reported to be transcriptional activators of FSH (Table 1, Supplementary Material). The data also shows SP1 as a potential key TF downstream of FSH in human GCs as it has SP1 binding sites with a very high similarity (Core similarity = 1) (Table 2). The increased binding of SP1 to IRS-2 promoter by FSH in human GCs was validated by ChIP assay (Fig. 2). Here, we have identified putative TFBS in the IRS-2 promoter region and data thereof was subjected to IRS-2-protein interaction analysis to emphasize the importance of this data. Unweighted binary interactions were analyzed for TF regulatory genes in IRS-2 network using Cytoscape software tool and R-code (Supplementary Material).

Table 1. Putative transcription factor binding sites in IRS-2 promoter region with match $\geq$ 15.

Matrix Family	Matrix Information	Match Total
ZF02	Zinc finger with KRAB and SCAN domains 3	88
ZF5F	Zinc finger / POZ domain transcription factor	73
KLFS	Kruppel-like factor 7 (ubiquitous, UKLF)	70
E2FF	E2F transcription factor 1	69
PLAG	Pleomorphic adenoma gene	56
MZF1	Myeloid zinc finger protein MZF1	50
TF2B	Transcription factor II B (TFIIB) recognition element	48
IKZF	IKAROS family zinc finger 5 (Pegasus)	44
SP1F	Specificity protein 1, ubiquitous zinc finger transcription factor	40
EGRF	EGR1, early growth response 1	39
SPZ1	Spermatogenic Zip 1 transcription factor	32
EBOX	E-box binding factors	29
AP2F	Transcription factor AP-2, alpha	28
MAZF	Myc associated zinc fingers	27
IKRS	Ikaros 2, potential regulator of lymphocyte differentiation	25
HESF	Drosophila hairy and enhancer of split homologue 1 (HES-1)	24
NDPK	Nucleoside diphosphate kinase	16
AP1R	Transcription factor AP-1	15

Highlighted in yellow are the transcription factors activated by FSH.

Table 2. SP1 binding sites identified in the human IRS-2 promoter region.

Detailed Matrix Information	Start position	End position	Anchor position	Strand	Core similarity	Matrix similarity	Sequence
SP1	-50	-66	-58	(-)	1.000	0.912	agcatGGGCggcgagcc
SP1	-76	-92	-84	(-)	1.000	0.877	ggcggGGGCtgcggcct
SP1	-102	-118	-110	(-)	1.000	0.997	gggcgGGGCgggggatc
SP1	-107	-123	-115	(-)	1.000	0.968	cggctGGGCggggcggg
SP1	-118	-134	-126	(-)	1.000	0.873	gggcgGGGCcgcggctg
SP1	-123	-139	-131	(-)	1.000	0.966	gcgccGGGCggggccgc
SP1	-207	-223	-215	(-)	1.000	0.980	gggcgGGGCgggccacg
SP1	-212	-228	-220	(-)	1.000	0.990	aagagGGGCggggcggg
SP1	-237	-253	-245	(-)	1.000	0.924	acccaGGGCgggaaaag
SP1	-298	-314	-306	(+)	1.000	0.855	ggccgGGGCcgccccac
SP1	-300	-316	--308	(-)	1.000	0.926	gggtgGGGCggccccgg
SP1	-310	-326	-318	(-)	1.000	0.950	cggccGGGCggggtggg
SP1	-321	-337	-329	(+)	1.000	0.888	cggccGGGCcggggcct
SP1	-382	-398	-390	(+)	1.000	1.000	aagggGGGCggggcggg
SP1	-387	-403	-395	(+)	1.000	0.997	gggcgGGGCgggggcgc
SP1	-399	-415	-407	(-)	1.000	0.856	ggggcGGGCcgcgcgcc
SP1	-403	-419	-411	(-)	1.000	0.980	cgcggGGGCgggccgcg
SP1	-479	-495	-487	(+)	1.000	1.000	cagcgGGGCggggcggc
SP1	-484	-500	-492	(+)	1.000	0.919	gggcgGGGCggccgcgc
SP1	-753	-769	-761	(-)	1.000	0.887	ccgcgGGGCcgagccta
SP1	-782	-798	-790	(-)	1.000	0.886	tcgctGGGCcgggagtc
SP1	-808	-824	-816	(-)	1.000	0.921	ctctcGGGCggcgccgg
SP1	-864	-880	-872	(-)	1.000	0.920	ggcgcGGGCggtggccg
SP1	-987	-1003	-995	(+)	1.000	0.927	tgatcGGGCgggcggcc
SP1	-991	-1007	-999	(+)	1.000	0.894	cgggcGGGCggccgggc
SP1	-1025	-1041	-1033	(-)	1.000	0.963	ggcgcGGGCgggggcgg
SP1	-1040	-1056	-1048	(-)	1.000	0.944	gcgagGGGCggaggggg

Start/End position, starting/ending position of the consensus binding site in the sequence (relative to IRS-2); Core similarity, core consensus sequence (4 highest conserved positions) similarity factor (0–1); Matrix similarity; matrix (groups of functionally similar transcription factors) similarity factor (0–1); SP1/GC, stimulating protein 1/GC box elements.

4. Experimental design-materials and methods

4.1. Transcription factor binding sites analysis

We carried out an in-depth computational analysis of the transcription binding sites on human IRS-2 promoter to identify the potential TFs that are responsible for FSH mediated regulation of IRS-2 expression in human GCs. Human IRS-2 promoter sequence [3] was analysed for putative TFBS using MatInspector software version 8.1, Matrix Library 9.1 from the Genomatix suite$_{v3.4}$ [1]. The parameters for comparing the binding sites with data base were set at matrix similarity and core similarity 0.85 (maximum 1.00) (Supplementary Material). TFBS in the IRS-2 promoter region were explored for IRS-2-protein interactions using open-access database [4] for experimentally verified human transcriptional regulation interactions (HTRIdb) (Supplementary Material).

4.2. Cell culture

Human GCs were isolated from the follicular fluid aspirates obtained after IVF treatment of subjects as described earlier [5]. Briefly, the follicular fluid was centrifuged at 350 × g for 15 min to pellet follicular cells and the GCs were isolated on Percoll gradient. The cells were seeded in DMEM containing 5% FBS and supplemented with 1x antibiotic antimycotic solution in multiwell plates at 37°C in 5% CO_2.

4.3 Chromatin Immunoprecipitation (ChIP) and reverse-transcription quantitative PCR (qRT-PCR)

Cells were serum starved and left either in the basal condition or with FSH (25 ng/mL) for 2 h. ChIP assay was performed as described earlier [6-7]. Briefly, cells were fixed with 1% formaldehyde, quenched with 0.125 M glycine and washed with cold PBS. After cell lysis, protein-DNA complexes were incubated with 2 μg of anti-SP1 and 2 μg non-specific IgG (normal rabbit IgG, Santa Cruz) antibodies and protein G agarose. 50 μl of cell lysate was used as input control. Protein-DNA complexes were released from the antibodies with elution buffer. After RNase A treatment, each sample was decrosslinked at 67°C overnight and DNA was purified using chromatin IP DNA purification kit (Active Motif, Carlsbad, CA) and then subjected to semi-quantitative PCR analysis with specific primers for SP1 binding site on promoter of IRS-2 gene. Primer sequences for IRS-2 promoter were 5'-ACAAGCCGCTGATTAATGAGGC-3' and 5'-TGACTCGGCGTTACGC AGGCAC-3'. Relative real-time qPCR was performed on Applied Biosystems 7500 Fast Real Time PCR System using a Power SYBR® Green PCR Master Mix. PCR amplified products were separated on 1.5% agarose gel in TAE buffer. The quantity of mRNA was calculated based on the cycle threshold (Ct) values which were normalized to the expression of the reference gene (β2M), which served as internal control. Data are expressed as % input after normalizing the Ct values obtained from SP1 antibody treated samples with the Ct values from input DNA. All experiments were performed in triplicates.

Acknowledgements

We are thankful to Avijit Podder for the analysis of IRS-2-protein interactions. This work was supported by research grant (Ref. no. BT/PR 5379/MED/14/631/2004; BT/PR8330/AAQ/01/313/2006) from the Department of Biotechnology (DBT), Government of India, New Delhi, India to RS.

References

[1] K. Cartharius, K. Frech, K. Grote, B. Klocke, M. Haltmeier, A. Klingenhoff, M. Frisch, M. Bayerlein, T. Werner, MatInspector and beyond: promoter analysis based on transcription factor binding sites, Bioinformatics 13 (2005) 2933-2942.

[2] Anjali G., S. Kaur, R. Lakra, J. Taneja, G.S. Kalsey, A. Nagendra, T.G. Shrivastav, M. Gouri Devi, N. Malhotra, A. Kriplani and Rita Singh, FSH stimulates IRS-2 expression in human granulosa cells through cAMP/SP1, an inoperative FSH action in PCOS patients, Cellular Signalling, 27, 2452-2466, 2015.

[3] L. Vassen, W. Wegrzyn, L. Klein-Hitpass, Human insulin receptor substrate-2: gene organization and promoter characterization, Diabetes 48 (1999) 1877–1880.

[4] L.A. Bovolenta, M.L. Acencio, N. Lemke, HTRIdb: an open-access database for experimentally verified human transcriptional regulation interactions, BMC Genomics. 13: (2012), 405-415.

[5] S. Kaur, K.J. Archer, M.G. Devi, A. Kriplani, J.F. 3rd Strauss, R. Singh, Differential gene expression in granulosa cells from polycystic ovary syndrome patients with and without insulin resistance: identification of susceptibility gene sets through network analysis, J. Clin. Endocrinol. Metab. 97 (2012) E2016-E2021.

[6] M. Udelhoven, M. Pasieka, U. Leeser, W. Krone, M. Schubert, Neuronal insulin receptor substrate 2 (IRS2) expression is regulated by ZBP89 and SP1 binding to the IRS2 promoter, J. Endocrinol. 204 (2010) 199–208.

[7] M. Udelhoven, U. Leeser, S. Freude, M.M. Hettich, M. Laudes, J. Schnitker, W. Krone, M. Schubert, Identification of a region in the human IRS2 promoter essential for stress induced transcription depending on SP1, NFI binding and ERK activation in HepG2 cells, J. Mol. Endocrinol. 44 (2010) 99-113.

†Corresponding Author

Address: Division of Molecular Endocrinology and Reproduction, Department of Zoology, University of Delhi, Delhi-110007, India

E-mail: ghrika_s@yahoo.com, ritas@zoology.du.ac.in

Telephone: +91-8527820707, 91-11-27667985/308

Figure

Figure 1

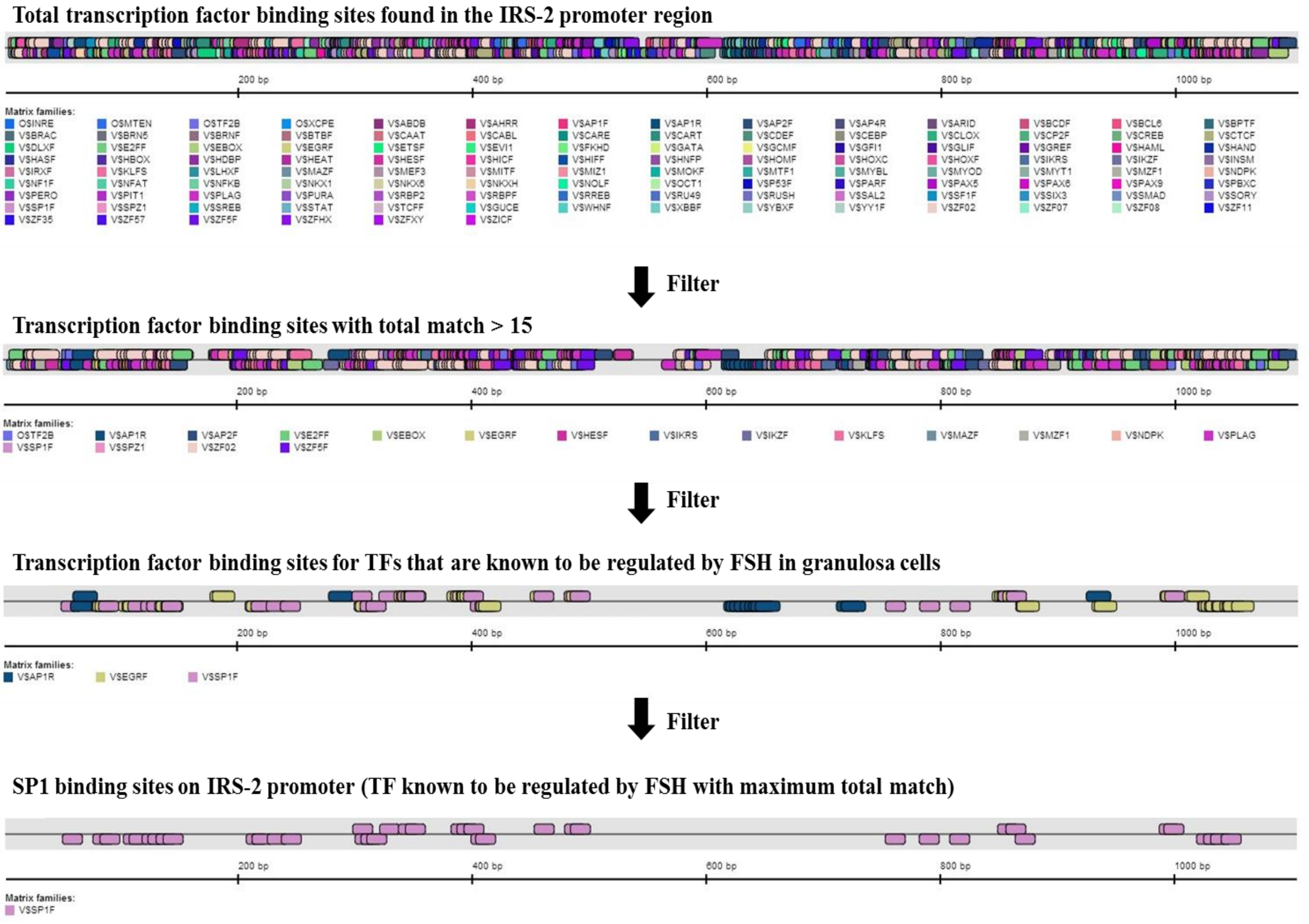

Figure 2

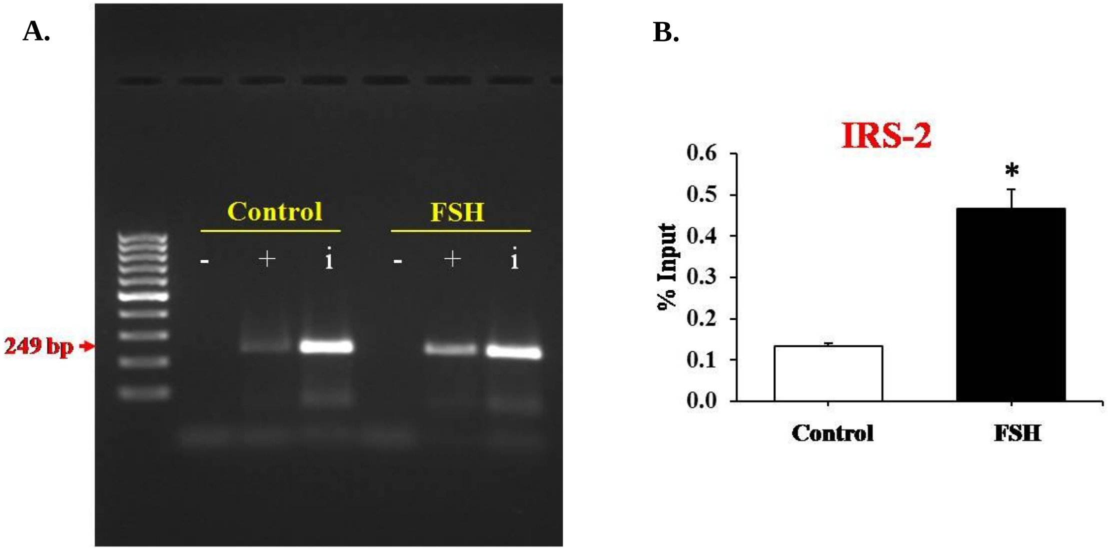
A.
Control
FSH
- + i - + i
249 bp
B.
IRS-2
% Input
0.6
0.5
0.4
0.3
0.2
0.1
0.0
*
Control
FSH

Figure 3

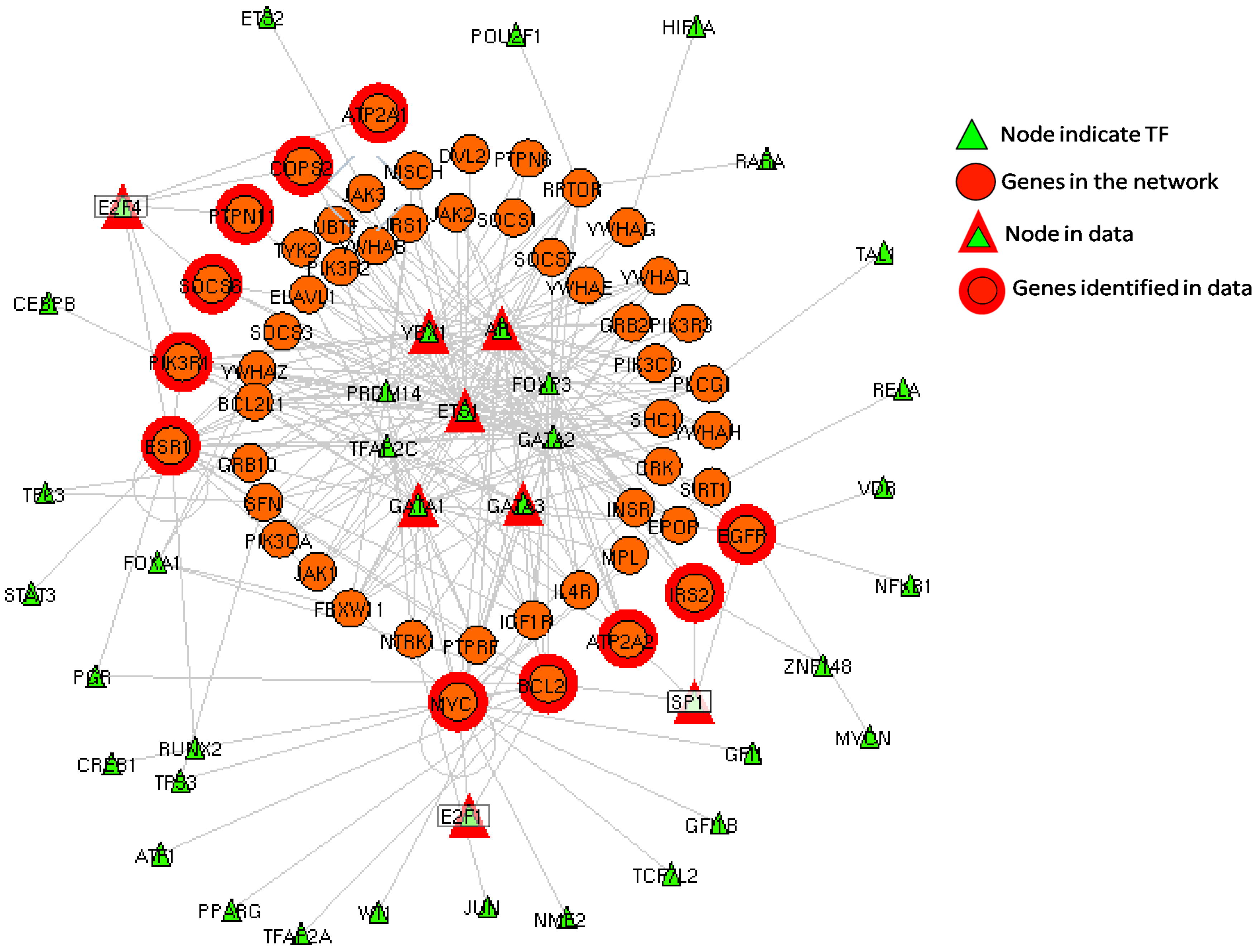

Node indicate TF
Genes in the network
Node in data
Genes identified in data
E2F4
E2F1
SP1
MYC
BCL2
EGFR
IRS2
ESR1
ATP2A1
ATP2A2
JAK2
JAK1
SOCS3
NTRK1
IL4R
MPL
SIRT1

Figure captions

Fig. 1. Identification of putative transcription factor-binding sites in the IRS-2 promoter region using Genomatix MatInspector software.

Fig. 2. FSH increases SP1 binding to IRS-2 promoter in human granulosa cells. ChIP assay was performed for verification of SP1 binding to IRS-2 promoter. Input reflects the relative amounts of sonicated DNA fragments before immunoprecipitations. (A) The relative amounts of IRS-2 promoter DNA fragments were determined with semi-quantitative PCR by separating the amplified product on 1.5% agarose gel. (B) The relative amounts of IRS-2 promoter DNA fragments were determined with qRT- PCR. Data are expressed as % input after normalizing the Ct values obtained from SP1 antibody treated samples with the Ct values from input DNA. Values presented are mean ± SD from 3 independent experiments (n = 3). *$P < 0.05$ vs. untreated. – IgG as negative control; + SP1 Antibody; i Input (Total sonicated DNA) as positive control.

Fig. 3. TF regulatory gene network for IRS-2.

*Conflict of Interest Form

Click here to download Conflict of Interest Form: coi_disclosure.pdf

Supplementary material

Click here to download Supplementary material: Supplementary data.zip
